# The complete genome sequence of *Caulobacter* phages Senya and Shash

**DOI:** 10.1128/mra.00457-26

**Published:** 2026-07-16

**Authors:** Enya M. Hoxha, Gillian D. Brown, Kaylyn A. Niemiec, Jolene Ramsey

**Affiliations:** 1 Department of Biology, Texas A&M University 14736, College Station, Texas, USA; 2 Center for Phage Technology, Texas A&M University 14736, College Station, Texas, USA; University of Pittsburgh School of Medicine, Pittsburgh, Pennsylvania, USA

**Keywords:** bacteriophages, *Caulobacter*, genomics

## Abstract

Newly discovered *Caulobacter* phages Senya and Shash are temperate siphophages infecting *C. crescentus* and *C. henricii*. Their genomes of size 225,427 and 223,261 bp with 38 and 33 tRNAs, respectively, encode host takeover genes, including GcrA-like cell cycle regulators.

## ANNOUNCEMENT

*Caulobacter crescentus* (taxonomic name *Caulobacter vibrioides*) is an environmental Gram-negative bacterial species that performs asymmetric cell division to produce motile swarmer cells from sessile stalked cells (1–3). Its relative, *Caulobacter henricii,* remains an understudied stalked bacterium (4, 5). Here, new *Caulobacter* phages Senya and Shash are described.

**Fig 1:**
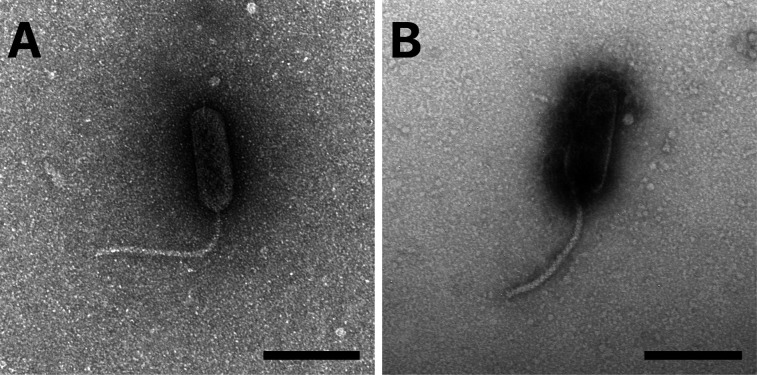
Transmission electron micrographs of bacteriophages (**A**) Senya and (**B**) Shash. Both phages display siphovirus morphology with flexible tails and prolate capsids. The scale markers each represent 200 nm.

Phages Senya and Shash were isolated from stagnant pond water collected in College Station, TX (GPS coordinates 30º32′48.00″N, 96º16′58.90″W) and Navasota, TX (GPS coordinates 30º23′21.23″N, 96º03′58.88″W), respectively. After ambient temperature transport, samples were filtered through 0.22 µm polyethersulfone and stored between 4 and 8°C. Thirty-milliliter aliquots were enriched with saturated *C. henricii* CB4 (ATCC 15253) cultures. Senya and Shash phages also plaqued on *C. crescentus* CB15 (ATCC 19089). Both hosts were grown to saturation on peptone yeast extract (PYE) medium with the addition of 1.5% calcium chloride at 27 and 30°C, respectively. Isolated plaques underwent sterilization with 2% chloroform during three rounds of purification by the soft-agar overlay method (6). Lysate samples were negatively stained with 2% uranyl acetate and observed using a transmission electron microscope at the Texas A&M Microscopy and Imaging Center (Figure 1). Genomic DNA was purified using the Promega Wizard DNA clean-up system as previously described (7). SeqCoast Genomics (Portsmouth, NH) used an Illumina DNA Prep Tagmentation Kit for library preparation. The Illumina NextSeq 2000 platform with a 300-cycle flow cell kit sequenced the unique dual indexes with 2 × 150 bp reads. Reads from 882,226 (Senya) and 923,555 (Shash) spots were subject to quality control using FastQC v0.11.9 (https://www.bioinformatics.babraham.ac.uk/projects/fastqc/). The contiguous genome was assembled from these reads using Shovill v1.1.0 at 29.8× (Senya) and 30.2× (Shash) coverage (8). These contigs were then closed by PCR, confirmed by Sanger sequencing, and reopened following the example of other phiCbk-like phages (9). Senya has 9,269 bp direct terminal repeats, while Shash has 11,098 bp repeats. The reopened genomes were annotated using the 2024 Galaxy release 24.2 and Apollo 4.2.13 pipeline at https://phage.usegalaxy.eu/ (10–12). GLIMMER v3 and MetaGeneAnnotator v1.0 were used for protein-coding gene detection, and ARAGORN v2.36 was used for tRNA detection (13–15). Functional prediction used BLAST v2.9.0, TMHMM v2.0 for transmembrane domain identification, and InterProScan v5.33 for conserved domain searches (16–18). Supporting analysis was completed using HHpred, Foldseek, and Alphafold3 (19–21). All tools used default settings.

Siphophages Senya and Shash (GenBank PX223409, PX223408) genome characteristics are summarized in Table 1. By BLASTn, they share 81.1% nucleotide identity between them. Senya is 79.8% identical at the nucleotide level with *Caulobacter* phage CcrRogue (GenBank NC_019408), but Shash shares 97.7% nucleotide identity with *Caulobacter* phage Magneto (GenBank JX100812). Notable characteristics of Senya and Shash include tyrosine integrases (GenBank YDS81042, YDS80702), suggesting they are temperate phages (22), and the presence of GcrA-like cell cycle regulators (GenBank YDS81106, YDS80791), which are σ70 cofactors involved in swarmer-to-stalked cell differentiation (23).

**TABLE 1:** Summary of Senya and Shash genomic characteristics

Genomic characteristics	Senya	Shash
Genome size (bp)	225,427	223,261
GC content (%)	65	66
Protein-coding genes	335	329
Protein-coding genes assigned predicted functions	57	49
tRNAs	38	33
tmRNAs	0	1

## Data Availability

The genomic sequence and associated data for Senya and Shash have been deposited under GenBank accession numbers PX223409 and PX223408, BioProject PRJNA222858, Sequence Read Archive entries SRR35158189 and SRR35158190, and BioSample accessions SAMN50750707 and SAMN50750706, respectively.
